# Secondary bile acids: an underrecognized cause of colon cancer

**DOI:** 10.1186/1477-7819-12-164

**Published:** 2014-05-24

**Authors:** Hana Ajouz, Deborah Mukherji, Ali Shamseddine

**Affiliations:** 1 Department of Hematology/Oncology, American University of Beirut Medical Center, PO Box 11-0236, Riad El Solh, Beirut 1107 2020, Lebanon

## Abstract

Bile acids were first proposed as carcinogens in 1939. Since then, accumulated evidence has linked exposure of cells of the gastrointestinal tract to repeated high physiologic levels of bile acids as an important risk factor for gastrointestinal cancers. High exposure to bile acids may occur in a number of settings, but most importantly, is prevalent among individuals who have a high dietary fat intake.

A rapid effect on cells of high bile acid exposure is the generation of reactive oxygen species and reactive nitrogen species, disruption of the cell membrane and mitochondria, induction of DNA damage, mutation and apoptosis, and development of reduced apoptosis capability upon chronic exposure. Here, we review the substantial evidence of the mechanism of secondary bile acids and their role in colon cancer.

## Introduction

Bile Acids (BA) are normal components of the lumenal contents of the gastrointestinal (GI) tract, where they enable absorption of lipids, cholesterol, and fat-soluble vitamins. In essence, they act as a physiologic detergent and regulator of intestinal epithelial homeostasis in the gastrointestinal tract [1].

However, BAs, specifically lithocholic acid (LCA) - a secondary BA - also constitute a rare example of toxic endobiotics [2]. In fact, BAs were first proposed as a potential tumor-promoting agent in 1939 [3].

At high physiologic concentrations, BAs can cause oxidative/nitrosative stress, DNA damage, apoptosis, and mutation [4]. Furthermore, frequently repeated and prolonged exposure of tissues to high physiological levels of BAs can lead to the generation of genomic instability, development of apoptosis resistance and, ultimately, cancer [4].

And since BAs are normal components of the luminal contents of the GI tract, finding the exact mechanism of their carcinogenic effect has become intriguing. Several factors have been found to increase levels of BAs: most importantly, a high dietary fat intake.

Our aim is to explain the correlation between the concentration of fecal secondary BAs -mainly deoxycholic acid (DOC) and LCA - and the colorectal cancer incidence that was highlighted by several epidemiological studies but whose molecular mechanism remain far from clear.

Furthermore, BAs were also found to be etiologic agents of other GI tract cancers, namely that of the esophagus [5], stomach [6], small intestine [7], liver [8], pancreas [9] and biliary tract [10].

## Review

### Biochemistry and physiology of secondary bile acids in the body

Primary BAs (cholic acid and chenodeoxycholic acid) are derived from cholesterol by a sequence of enzymatic reactions occurring mainly in the liver. Synthesis of a full complement of BAs requires 17 individual enzymes and occurs in multiple intracellular compartments that include the cytosol, endoplasmic reticulum (ER), mitochondria, and peroxisomes [11]. After synthesis, these BAs are conjugated with glycine or taurine and then excreted and stored in the gall bladder. In humans, BAs are largely re-absorbed in the terminal ileum by an active transport mechanism, but less than 5% of the BA pool enters the colon per day [12]. The BAs that enter the colon are metabolized by bacterial flora, where the primary BAs (cholic and chenodeoxycholic acid) are converted into the secondary BAs, DOC and LCA, respectively.

DOC is partly absorbed in the colon and enters the enterohepatic circulation, where it is conjugated in the liver and secreted in the bile [13]. LCA is fairly insoluble and little of it is reabsorbed [13]. Thus, the circulating BA pool (conjugated when it leaves the gallbladder, and then de-conjugated by action of bacterial enzymes after it enters the colon) is composed of about 30 to 40% each of cholic acid and chenodeoxycholic acid, about 20 to 30% of DOC, and less than 5% of LCA [14].

BAs are amphipathic and many of their properties are related to their amphipathic nature [15]. Their amphipathic nature enables them to get involved in emulsification and digestion of dietary fats; yet, levels above those that are physiologic are potentially membrane damaging.

### Factors that change secondary bile acids levels

Almost 40 years ago, Berg was the first to make the observation that CRC (colorectal cancer) risk was higher among descendants of individuals in low-risk populations after moving to developed countries and converting to a Western-type diet that is rich in red meat and saturated fatty acids [16]. This was also consistent with a prospective cohort study that investigated 55,487 Danish middle-aged men and women and suggested that adherence to recommendations for five lifestyle factors (physical activity, smoking, waist circumference, alcohol intake and diet) may indeed considerably reduce CRC risk [17].

Prolonged, high consumption of red meat and saturated fatty acids were found to increase CRC risk. In their case control study, Bayerdorffer *et al*. [18] confirmed that DOC (Doxycholic acid) levels were significantly higher in the sera of patients with colorectal adenomas. Later, Bayerdorffer *et al*. [19] found that this positive association between DCA in the serum and colorectal adenomas was highest in the unconjugated fraction, which originates directly from the colon. High fat diets stimulate bile discharge, hence they increase the concentration of BAs above physiologic levels [20]. Population-based studies have shown that subjects who consume high-fat and high-beef foods display elevated levels of fecal secondary BAs, mostly DOC and LCA, as do patients diagnosed with colonic carcinomas [12,13]. Results of such studies, however, are not very coherent due to difficulties in accurately measuring secondary BA levels in their different forms. Such incoherencies transpired because only levels of free LCA were measured, when most of LCA is in sulphated form. The increase in DOC and LCA reflects increased production of BAs in order to emulsify the increased level of dietary fat. Consequently, elevated secondary BA levels would alter the growth of intestinal epithelium, thus acting as tumor promoters [21]. Add to that, nicotine from smoking can interact synergistically in colon cells to increase oxidative stress and DNA damage [22].

Conversely, diets rich in vegetables and fruits are linked to a decreased CRC incidence. Dietary fibers (from vegetables and fruits) can bind to LCA and aid in its excretion in stool [23]; as such, fibers can protect against colon cancer. Not only fibers play a protective role, but vitamin D and high dietary Calcium supplementation also inhibits colon carcinogenesis induced by either high-fat diets or intrarectal instillation of LCA [24].

LCA activates vitamin D receptor, which may activate a feed-forward catabolic pathway that leads to the detoxification of LCA. Whereas, high dietary calcium leads to the formation of insoluble calcium soaps, this in turn decreases the concentration of free BAs in the intestinal lumen that ultimately may protect against formation of colon cancer [25].

### Secondary bile acids and the plasma membrane

An essential constituent of plasma membrane is cholesterol, which rigidifies the membrane and is an important structural component of membrane microdomains [26]. Due to the fact that BAs are cholesterol derivatives with detergent properties, BAs may alter the stability of the membrane lipid bilayer [27]. In fact, BAs with increased hydrophobicity have a greater capacity to perturb the structure of, or partly digest, cell membranes [28]. Secondary BAs (DOC and LCA) also increased paracellular permeability in a dose-related manner, with LCA exerting more potent effects than DOC [29]. When present in high concentrations, secondary BAs, cause unspecific cell membrane damage resulting in focal destruction of intestinal epithelium (Payne, 2008 [4]). Worse yet, subsequent repair mechanisms involving inflammatory reactions and hyperproliferation of undifferentiated cells would then increase the risk of transition into a precancerous state. Hyperproliferation of the colorectal mucosa is regarded as an early step in colorectal tumorigenesis [30].

In the colonic epithelium, high secondary BA concentrations induce cell proliferation by activating epidermal growth factor receptors (EGFRs) and post-EGFR/ERK (extracellular signal-regulated kinase) signaling [31]. In addition, BA-induced hyperproliferation can occur through the activity of protein kinase C (PKC), which can be activated downstream of the EGFR or by membrane perturbations [32].

### Bile acids and nuclear receptors

Nuclear receptors (NRs) are transcription factors that act as sensors of dietary and endogenous molecules, translating nutritional and hormonal stimuli into transcriptional programs [33]. Recently, it has become apparent that nuclear BA receptors FXR, vitamin D receptor (VDR) and pregnane X receptor (PXR)/steroid xenobiotic receptor (SXR) play an important role in protecting against carcinogenic effects of BAs by activating transcriptional programs aimed at coordinating the control of BA uptake, detoxification, and basolateral secretion [34]. FXR, a member of the nuclear receptor superfamily, responds to BAs as physiological ligands [35]. FXR has a key role in activating pathways that maintain BA homeostasis. FXR protects against intestinal tumorigenesis, possibly by a mechanism involving induction of apoptosis [36].

Vitamin D deficiency is a known major risk factor for colorectal cancer [37]. When vitamin D binds to VDR it functions as a transcription factor for many genes. In this manner vitamin D exerts profound antimitogenic and prodifferentiating effects on many normal and malignant cells including colon cancer cells [24]. Thus in Vitamin D insufficiency levels may not be high enough for effective regulation of cell growth and function. VDR functions as a receptor of secondary BAs such as LCA, and has a key role in activating a pathway that detoxifies LCA [37]. Similarly, the human xenobiotic receptor SXR and its rodent homolog PXR are BA receptors that when activated induce a response that detoxifies BA [38]. PXR promotes BA detoxification by activating BA metabolizing enzymes and transporters [39]. In both human colon cancer cells and normal mouse colon epithelium, PXR/SXR protects against oxidant induced apoptosis.

Interestingly, the enteric NR transcriptome has been found to be downregulated in both mouse and human models of CRC when the tissues are progressing from normal intestinal epithelia to dysplastic lesions [40]. This suggests a therapeutic and/or diagnostic potential for these transcription factors in CRC.

### Decreased HLA class I mRNA

Reduced or lost expression of HLA (human leukocyte antigen) antigens is seen in human colon carcinogenesis [41]. LCA was found to reduce the expression of HLA class I antigens on the surface of colon cells by 42% [42]. This dose-dependent reduction was specific for both the target genes and the chemical structure of LCA. Not only LCA, but DOC also - though to a lesser extent - decreased steady-state HLA class I mRNA levels. Data pertinent to the *in vivo* status of HLA in response to LCA, however, is still lacking.

### Bile acids and production of reactive oxygen species and reactive nitrogen species

One of the most important cytotoxic effects of BAs is the increased production of reactive oxygen/nitrogen species (ROS/RNS) [43]. Activation of several plasma membrane enzymes - such as NAD (P) H oxidases and phospholipase A2 - lead to the production of ROS [44]. This may also be induced by perturbations in the mitochondrial membrane. Increased production of ROS/RNS, can lead to increased DNA damage and thus, increased mutation. The production of ROS/RNS following BA exposure is likely to occur through multiple pathways involving disruptions of the cell membrane and mitochondria [43].

### Bile acids and NF-kB activation

The balance between whether BAs induce proliferation or cell death in colonic epithelial cells is finely tuned by activation of NF-kB. The ability of BAs to induce activation of NF-kB largely determines intestinal epithelium cell fate [45]. Closely related to the BA’s ability to induce ROS production and inflammation, the redox-sensitive transcription factor NF-kB is also important in many cellular processes such as inflammation and apoptosis [4]. Persistent activation of this factor (NF-kB) in the colon results in colitis and ultimately colon cancer [46]. ROS dependent and independent pathways can activate NF-kB thus meticulously regulating the balance between cell proliferation and demise.

A report has highlighted the mechanism of DOC-induced NF-kB activation in HCT116 cells by showing the significant correlation that binds NAD (P) H oxidase, Na+/K + −ATPase, cytochrome P450 isoform 1B1, calcium levels and the terminal mitochondrial respiratory complex IV [47].

### Secondary bile acids and DNA damage

BAs induce DNA damage in colon cells with oxidative DNA damage being a likely component [48]. Defective repair of oxidative DNA damage is linked to increased risk of colon cancer. ROS causes oxidative damage in DNA by disrupting the base excision repair pathway.

Genetic instabilities and chromosomal alterations are frequently found in CRC [49]. BAs can induce genomic instability in colonic epithelial cells through multiple mechanisms, including the disruption of mitosis (leading to aneuploidy), defects in spindle assembly checkpoints, oxidative DNA damage, cell cycle arrest at G1 and/or G2 along with improper alignment of chromosomes at the metaphase plate and multipolar divisions [50]. The numerous studies showing that BAs induce DNA damage in colon cells suggest that BAs may also induce mutation and genomic instability. DOC may induce K-ras mutations as well as aneuploidy and micronuclei formation [51].

### Secondary bile acids and cell death

BAs have the potential to induce cell death both through nonspecific detergent effects and receptor-mediated interactions [52]. Elevated DOC and LCA levels promote apoptosis primarily through activation of the intrinsic apoptotic pathway involving stimulation of mitochondrial oxidative stress, generation of reactive oxygen species (ROS), cytochrome C (cytC) release and activation of cytosolic caspases [53]. Yui *et al*. recently reported a biphasic effect of cytoprotection and induction of apoptosis by hydrophobic BAs in the HCT116 cell line [54], with induction of different cellular responses depending on BA concentrations. This has raised a probability that epithelial cells of the intestinal tract acquire resistance to apoptosis after chronic exposure to low concentrations of hydrophobic BAs [34]. In a physiological condition, epithelial cells are exposed to proapoptotic stimuli, causing DNA damage or oxidative stress, some of which act as tumor initiators. Apoptosis that occurs spontaneously *in vivo* prevents tumor incidence by eliminating damaged cells [55]. Several studies of colon cancer patients have shown that epithelial cells in areas of the colonic mucosa that do not contain the cancer itself have increased resistance to induction of apoptosis by DOC [56]. In addition, the expression of anti-apoptotic protein Bcl-xL is elevated in the colorectal mucosa adjacent to colorectal adenocarcinomas [57].

## Conclusions

Since the CRC incidence and mortality rates are expected to increase in the coming decades, in-depth understanding of the molecular and cellular mechanisms underlying CRC becomes crucial. Focusing on the role of secondary BAs in developing CRC would be of great significance since altering the risks that increase secondary BAs would alter the incidence of CRC. Not only that, but secondary BAs may become an effective screening marker. Secondary bile acids solve the puzzle of colorectal cancer because they sit at the crossroad of nutritional and hormonal signals modulating the tangled interactions between the environmental factors, such as diet, and the nuclear receptors such as VDR. The pivotal question that emerges from the current evidence is to specify what epigenetic abnormalities are required in the crypt stem cell to render them prone to secondary BA. The next challenge is to unravel how potential stem cell specific mutations render them more prone to environmental factors, which in turn, renders them more prone toward either normal or tumorigenic phenotypes.

## Abbreviations

BA: bile acids; CRC: colorectal cancer; DOC: deoxycholic acid; EGFRs: epidermal growth factor receptors; ER: endoplasmic reticulum ERK, extracellular signal-regulated kinase; GI: gastrointestinal; LCA: lithocholic acid; NR: nuclear receptors; PKC: protein kinase C; PXR: pregnane X receptor; RNS: reactive nitrogen species; ROS: reactive oxygen species; SXR: steroid xenobiotic receptor; VDR: vitamin D receptor.

## Competing interests

The authors declare that they have no competing interests.

## Authors’ contributions

HA drafted the manuscript, AS and DM participated in the design and coordination of the study. All authors read and approved the final manuscript.
